# Transcriptomic Signatures Associated with Doxorubicin Treatment in Liposarcoma Reveal Coordinated Regulatory Patterns

**DOI:** 10.3390/diseases14060219

**Published:** 2026-06-18

**Authors:** Anas Khaleel, Sara Khaleel, Ruqaya Mohammed Ahmed, Ahmad Al Athamneh, Nour Amin Elsahoryi, Ahmed S. A. Ali Agha

**Affiliations:** 1 Faculty of Pharmacy and Medical Sciences, University of Petra, Amman 11196, Jordan; 2 Department of Pharmacy, Faculty of Pharmacy, Al-Zaytoonah University of Jordan, Amman 11733, Jordan; s.malkawi@zuj.edu.jo; 3 Department of Pharmacy, Al-Ma’moon University, Baghdad 10012, Iraq; ruqaya.m.ahmed@almamonuc.edu.iq; 4 Department of Nutrition, Faculty of Pharmacy and Medical Sciences, University of Petra, Amman 11196, Jordan; ahmad.alathamneh@uop.edu.jo (A.A.A.); nour.elsahoryi@uop.edu.jo (N.A.E.); 5 School of Pharmacy, Department of Pharmaceutical Sciences, The University of Jordan, Amman 11942, Jordan

**Keywords:** liposarcoma, doxorubicin, transcriptomics, tumor microenvironment, regulatory networks, chemotherapy response, gene expression profiling

## Abstract

**Background/Objectives:** Liposarcoma is a heterogeneous soft tissue sarcoma in which anthracycline-based chemotherapy, including doxorubicin, remains a cornerstone of treatment for advanced disease. However, variable and often limited therapeutic responses highlight the need for improved understanding of disease-associated transcriptional adaptation under chemotherapeutic stress. In this study, a bioinformatics-driven transcriptomic analysis was performed to characterize gene expression alterations associated with doxorubicin treatment in liposarcoma using publicly available data from the Gene Expression Omnibus (GSE12972). **Results:** Differential expression analysis identified 365 significantly altered genes, including 164 upregulated and 201 downregulated transcripts in treated samples compared with untreated controls. Functional interpretation using Ingenuity Pathway Analysis identified transforming growth factor beta 1 (*TGFB1*), tumor necrosis factor (*TNF*), and *SMARCA4* as prominent predicted upstream regulators associated with transcriptional programs related to extracellular matrix remodeling, inflammatory and immune modulation, epithelial-to-mesenchymal transition-like states, and chromatin-mediated transcriptional plasticity. Enriched canonical pathways included Liposarcoma tumor microenvironment-associated signaling and fibrosis-related pathways, reflecting stromal activation and immune-related transcriptional changes. Notably, fibroblast growth factor 1 (*FGF1*) emerged as a supportive regulatory node linked to survival- and anti-apoptotic gene expression patterns. **Conclusions:** Collectively, these findings provide a disease-oriented, cross-subtype systems-level view of the transcriptional changes associated with doxorubicin treatment in liposarcoma. This work is intended as a hypothesis-generating framework that may inform future functional studies and integrative approaches aimed at understanding therapeutic response and disease progression.

## 1. Introduction

Liposarcoma, a malignancy arising from adipose tissue, accounts for approximately 15–20% of adult soft tissue sarcomas and represents a significant clinical challenge due to its biological heterogeneity and variable therapeutic response [1]. Surgical resection and radiotherapy remain the cornerstone treatments for localized disease; however, chemotherapy is frequently employed in advanced, metastatic, or unresectable cases [2]. Among available chemotherapeutic agents, doxorubicin is considered a first-line option and has demonstrated clinical efficacy in liposarcoma. Nevertheless, both intrinsic and acquired resistance to doxorubicin substantially limit its long-term effectiveness [3]. This therapeutic resistance underscores the need for a deeper understanding of the molecular and regulatory processes underlying liposarcoma progression and treatment response.

Advances in high-throughput transcriptomic profiling and bioinformatics have enabled systematic interrogation of gene expression alterations associated with cancer development, therapeutic adaptation, and resistance [4]. Analyses of differentially expressed genes (DEGs), together with pathway enrichment and upstream regulator inference, provide a powerful framework for identifying regulatory programs that shape tumor behavior and the tumor microenvironment [5,6]. In liposarcoma, several regulatory factors have emerged as critical modulators of oncogenic processes, including transforming growth factor beta 1 (*TGFB1*), tumor necrosis factor (*TNF*), and the chromatin remodeling factor *SMARCA4* [2,7,8]. These regulators are implicated in processes such as epithelial-to-mesenchymal transition, inflammatory signaling, immune modulation, and transcriptional plasticity, all of which are closely associated with tumor progression and therapeutic resistance.

Growing evidence suggests that chemotherapeutic stress does not act solely on tumor cells in isolation but instead induces transcriptional changes consistent with microenvironment-related gene programs across the broader tumor microenvironment, influencing stromal activation, extracellular matrix remodeling, immune cell dynamics, and adaptive transcriptional states. Transcriptomic analyses capturing these coordinated changes offer insight into how regulatory programs converge to support tumor survival under treatment pressure. However, translating complex bioinformatic outputs into an integrated biological narrative remains a key challenge.

In this study, publicly available transcriptomic data from the Gene Expression Omnibus (GEO) were analyzed using Ingenuity Pathway Analysis (IPA) to identify DEGs, upstream regulators, and enriched canonical pathways associated with doxorubicin-treated liposarcoma samples [9]. The primary objective was to characterize regulatory programs linked to therapeutic adaptation, with particular emphasis on *TGFB1*-, *TNF*-, and *SMARCA4*-associated transcriptional networks, as well as their relationship to tumor microenvironment remodeling and immune-related processes.

**Figure 1:**
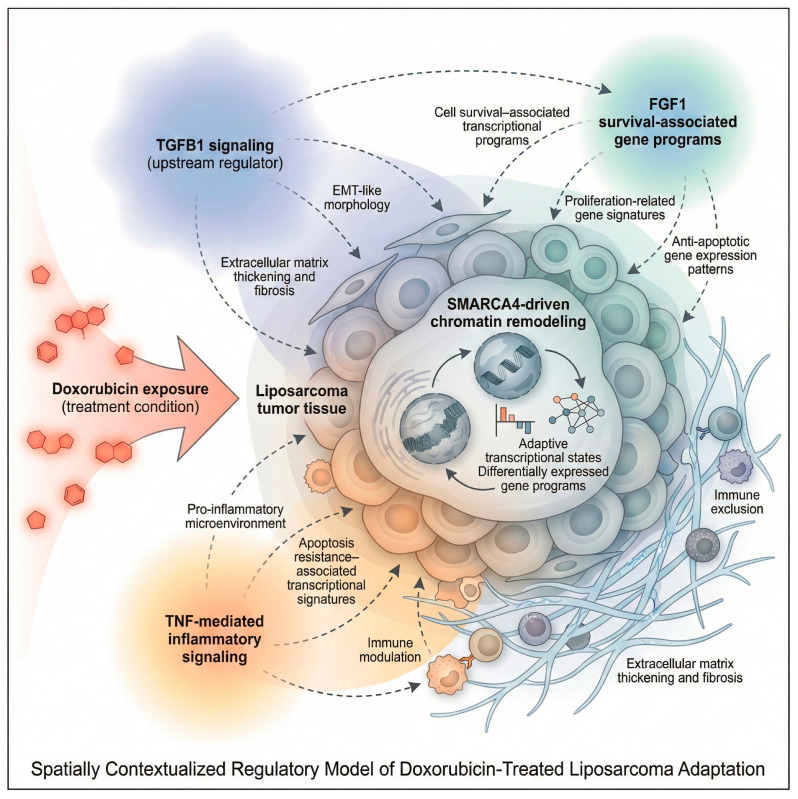
Spatially contextualized regulatory model of doxorubicin-treated liposarcoma. This schematic summarizes upstream regulatory programs inferred from transcriptomic and pathway analyses of doxorubicin-treated liposarcoma. *TGFB1*, *TNF*, and *SMARCA4* are highlighted as key upstream regulators associated with extracellular matrix remodeling, inflammatory and immune-related transcriptional signatures, and chromatin remodeling-driven transcriptional plasticity, respectively. *FGF1* is shown as a supportive node linked to survival- and proliferation-associated gene programs. Doxorubicin is depicted as a treatment condition influencing these regulatory programs. All elements represent inferred transcriptional associations and do not indicate experimentally validated mechanisms or causal relationships.

To orient the reader and provide a conceptual framework for the analyses performed, we present a spatially contextualized regulatory model summarizing the upstream regulators and transcriptional programs investigated in this study (Figure 1).

This model serves as an integrative, hypothesis-generating overview derived from transcriptomic and pathway analysis, rather than a representation of experimentally validated molecular mechanisms.

## 2. Materials and Methods

### 2.1. GEO Database Website

The gene expression data were downloaded from the Gene Expression Omnibus (GEO) repository (GPL96 platform; GSE12972, Daigeler et al., 2008 [10]).

The dataset consists of 38 microarray samples: each is a doxorubicin-treated array in the experimental group, paired with a vehicle-treated (PBS) control array for each of 19 primary human liposarcoma cultures, resulting in a fully paired experimental design. Tumor cells were isolated from the surgical specimens of patients who underwent surgical resection of histopathologically confirmed liposarcoma, enzymatically dissociated, purified by Ficoll–Hypaque gradient, and short-term cultured in Leibovitz’s L-15 medium with 2 mM glutamine and 10% fetal bovine serum. Cultures at subconfluence were treated with doxorubicin (0.5 μg/mL) or with an equal volume of PBS for 24 h before RNA extraction, and hybridization was performed. The study used doxorubicin alone in vitro, so the observed changes in transcription are not complicated by the clinical combination therapies (e.g., ifosfamide, dacarbazine) that routinely treat sarcomas.

The cohort is representative of the main liposarcoma sub-types and grades. Histologically, there were 7 well-differentiated/atypical lipoma, 4 dedifferentiated, 4 pleomorphic, 3 myxoid/round cell, and 1 myxoid liposarcoma; they were spread among G1 (*n* = 4), G2 (*n* = 8), and G3 (*n* = 7). Of the 16 tumors, 12 were from primary tumors, six from local recurrences and one from a metastatic deposit, and their maximal tumor diameters ranged from 1.0 to 38.5 cm. In the original study, samples were also divided into high (>2000 DEGs), intermediate (100–1000 DEGs) and low (<100 DEGs) responders depending on the range of transcriptional response to doxorubicin, which had a strong correlation with grade, with all G3 samples being high and most G1 samples being low responders. The original GEO record and publication report the ethics approvals granted at the institution of origin.

Given that the data are from short-term primary cultures rather than intact tumor tissue, interpretations of the immune and stromal signatures are presented as inferred transcriptional associations, which may reflect the presence of residual non-malignant cells co-cultured with the dissociated specimens, rather than as measurements of an intact tumor microenvironment. This restriction is discussed in more detail in Section 4.

### 2.2. Identification of Differentially Expressed Genes (DEGs)

Differential expression was carried out in GEO2R (GEO web interface), and the limma R package (version 3.66) is the log_2_-transformed and quantile-normalized expression matrix GSE12972. Samples were then split into two categories: doxorubicin-treated (*n* = 19) and vehicle-treated control (*n* = 19). The original experiment was performed as a paired design (treated array to a control array from the same primary culture), so a paired contrast was specified in the GEO2R model. Empirical Bayes shrinkage was performed on the standard errors, and linear models were fit on a gene-by-gene basis. Multiple-testing correction was performed using the Benjamini–Hochberg false discovery rate procedure, and genes with a *p*-value < 0.05 were deemed as being either up- or down-regulated. No additional fold-change cut-off was used, as per the default in GEO2R, to maintain sensitivity for moderate-effect transcripts in downstream pathway inference. This resulted in 365 DEGs (164 up and 201 down). Log_2_ fold-change, raw *p*-value, BH-adjusted *p*-value and Affymetrix probe ID were preserved for each DEG. When several probes were associated with the same gene symbol, the probe with the lowest adjusted *p* value was chosen to represent that gene symbol for subsequent analyses. No arrays were excluded because all 38 passed GEO2R’s built-in quality assessment (value-distribution diagnostics and boxplot of normalized intensities).

#### Batch and Confounding Considerations

Because the GSE12972 set was collected in a single laboratory and processed on a single microarray platform (Affymetrix HG-U133A, GPL96) using a uniform protocol with minimal technical batch effects, it was chosen for further analysis. The paired design removes the inter-patient biological variability (e.g., tumor grade, subtype, age, sex, anatomical site) and allows us to isolate the transcriptional response to doxorubicin exposure within each primary culture. No surrogate variable analysis or ComBat correction was performed (neither is suitable for a paired single-batch design; corrections may also remove the biological signal of interest).

### 2.3. Pathway and Network Analysis Using IPA

The 365 DEGs were uploaded to the QIAGEN Ingenuity Pathway Analysis (IPA; QIAGEN, Redwood City, CA, USA) software (QIAGEN; Ingenuity Knowledge Base current at the time of analysis). The input file was a list of genes at the gene level, containing HGNC gene symbols, log_2_FC values, and BH-adjusted *p*-values. A Core Expression Analysis was done using the following parameters: Reference Set: Ingenuity Knowledge Base (Genes Only); Relationships: Direct and Indirect; Interaction Networks: limited to 25 networks per analysis and 35 molecules per network; Data Sources: all sources in the Knowledge Base; Confidence: Experimentally Observed and High (Predicted); Tissues and Cell Lines: All (no restriction); Mutations: All. The default activation z-score algorithm was employed for Upstream Regulator Analysis, and regulators were deemed significant when |z| ≥ 2 and the overlap *p*-value was <0.01. Pathways were enriched using a right-tailed Fisher’s exact test, and those with BH-adjusted *p* < 0.05 were retained, with activation states being determined by the z-score when the IPA-defined pattern was adequate. Networks were analyzed using the default scoring in IPA, –log_10_ of the right-tailed Fisher’s exact test *p*-value for the network.

Upstream regulator analysis was conducted to predict transcriptional regulators whose activity could explain the observed expression patterns. Canonical pathway enrichment was assessed using right-tailed Fisher’s exact test, and pathway activation states were inferred using IPA’s Z-score algorithm, where applicable. Network analysis was used to visualize relationships among DEGs and to identify functionally related gene clusters associated with tumor microenvironment remodeling, immune regulation, and adaptive transcriptional programs.

**Figure 2:**
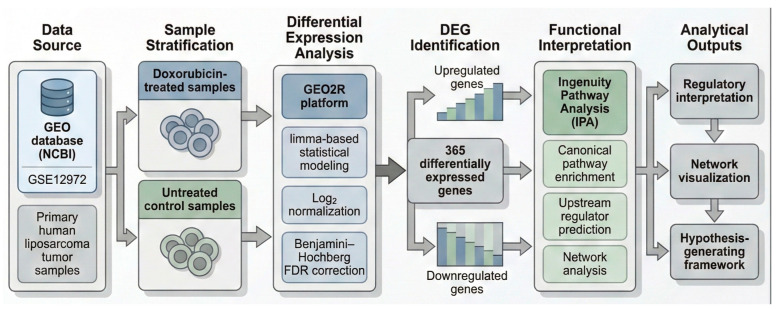
Bioinformatics workflow for transcriptomic analysis of doxorubicin-treated liposarcoma. Gene expression data from the GEO database (GSE12972) were analyzed using GEO2R for differential expression analysis, followed by functional interpretation using Ingenuity Pathway Analysis (IPA).

An overview of the transcriptomic analysis workflow employed in this study is summarized in Figure 2.

## 3. Results

This study identified key transcriptional changes across the four major liposarcoma subtypes represented in the GSE12972 dataset and regulatory networks associated with doxorubicin-treated liposarcoma using differential gene expression and pathway analyses. A total of 365 differentially expressed genes (DEGs) were identified, including 164 upregulated and 201 downregulated genes in doxorubicin-treated samples compared with untreated controls. These DEGs were further analyzed using Ingenuity Pathway Analysis (IPA) to identify enriched canonical pathways, upstream regulators, and gene interaction networks associated with the transcriptional response to treatment.

### 3.1. Identification of Key Upstream Regulators

Upstream regulator analysis identified *TGFB1*, *TNF*, and *SMARCA4* as the most significantly predicted activated upstream regulators, with Z-scores of 5.3, 5.0, and 4.6, respectively. The top ten predicted upstream regulators are summarized in Table 1.

**Figure 3:**
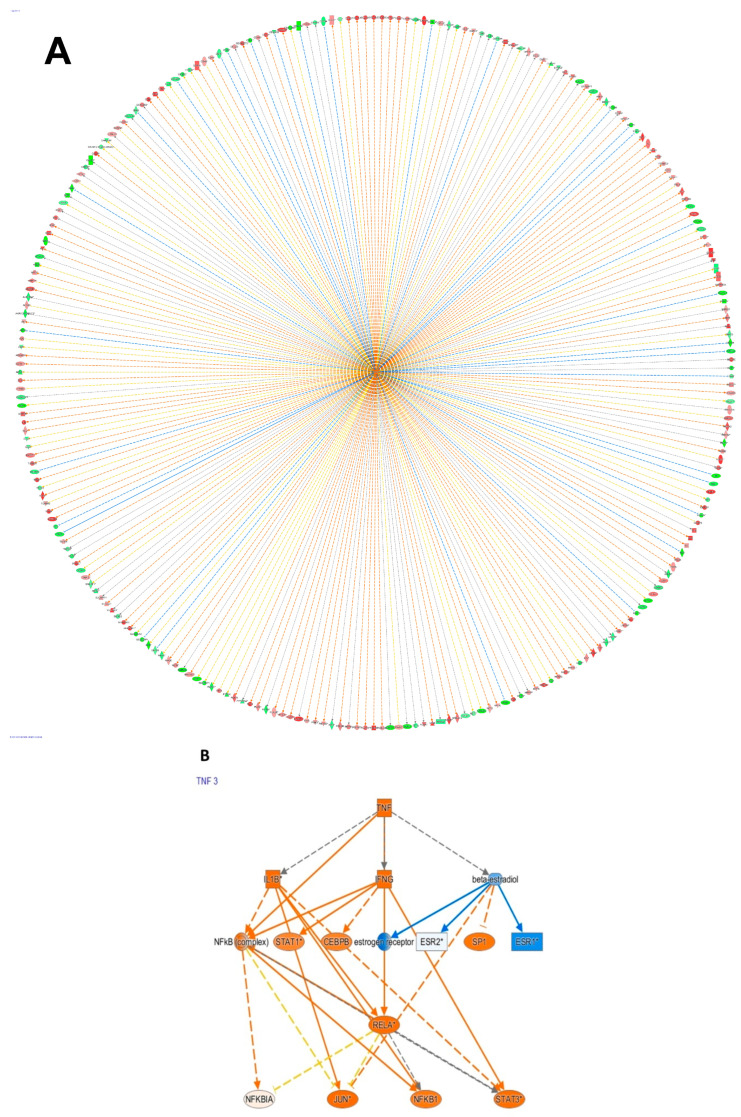
Upstream regulators *TGFB1* (**A**) and *TNF* (**B**) identified in liposarcoma with predicted significant stimulation (*TGFB1*: *p* = 2.43 × 10^−6^, Z = 5.3; *TNF*: *p* = 3.24 × 10^−30^, Z = 5.03). Red/green denotes over-/under-expression, while blue/orange represents inhibition/activation. Solid lines indicate direct interactions, dashed lines denote indirect relationships, and dotted lines suggest machine-learning-derived associations. (*) Asterisks indicate multiple identifiers (e.g., probe sets) mapping to a single gene or molecule in the Global Molecular Network.

To further visualize upstream regulator activation patterns, network representations of *TGFB1* and *TNF* were generated using IPA. These networks illustrate predicted regulatory relationships between the upstream regulators and their downstream transcriptional targets (Figure 3).

### 3.2. TGFB1-Associated Canonical Pathways

**Figure 4:**
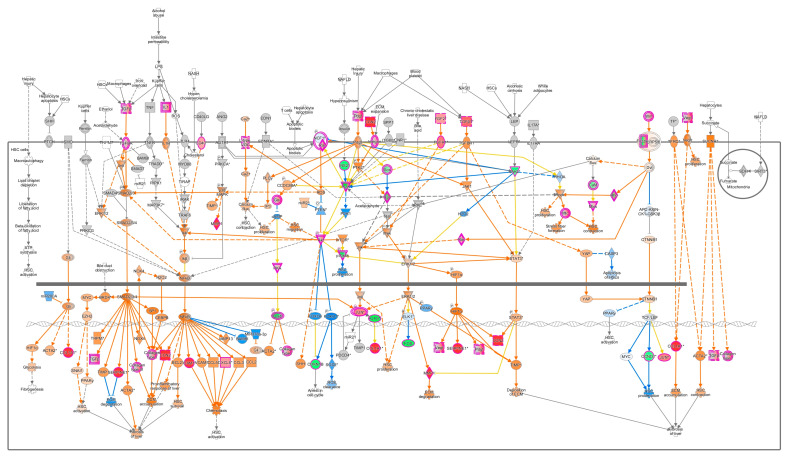
Hepatic fibrosis signaling was identified as one of liposarcoma’s top predicted canonical pathways. Red/green denotes over-/under-expression, and blue/orange denotes inhibition/activation. Purple color nodes reflect IPA’s predefined canonical-pathway template coloring (molecule class within the pathway) Solid lines show direct interactions, dashed lines represent indirect interactions. (*) Asterisks indicate multiple identifiers (e.g., probe sets) mapping to a single gene or molecule in the Global Molecular Network.

Canonical pathway analysis revealed hepatic fibrosis signaling and tumor microenvironment-related pathways among the most significantly enriched pathways associated with predicted *TGFB1* activation. These pathways were characterized by differential expression of genes involved in extracellular matrix organization and stromal remodeling, as illustrated in Figure 4.

### 3.3. Global Regulatory Network Overview

**Figure 5:**
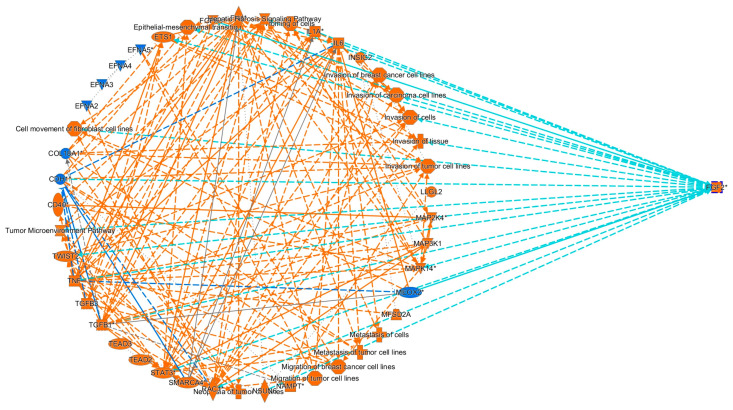
Graphical summary illustrating the predicted activation and suppression patterns of key regulators, with *TGFB1* and *TNF* identified as central network components, where blue and orange denote inhibition and activation, respectively. Solid lines show direct interactions, dashed lines represent indirect interactions. (*) Asterisks indicate multiple identifiers (e.g., probe sets) mapping to a single gene or molecule in the Global Molecular Network.

A graphical summary was generated to provide an overview of the activation and inhibition states of key upstream regulators identified in the analysis. This network highlights *TGFB1* and *TNF* as central regulatory nodes within the broader transcriptional landscape (Figure 5).

**Figure 6:**
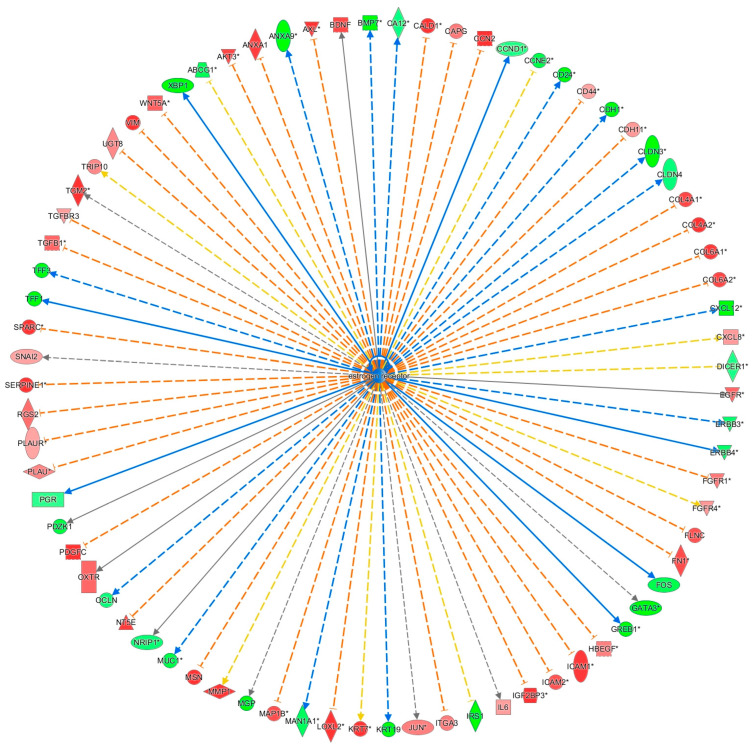
The estrogen receptor is predicted to be inhibited in liposarcoma (*p* = 3.7 × 10^−37^, Z = −5.8). Shapes indicate molecular class; red/green represent down-/up-regulation, and blue/orange indicate inhibition/activation. Red/green denotes over-/under-expression, and blue/orange denotes inhibition/activation. Solid lines show direct interactions; dashed lines represent indirect interactions (*). Asterisks indicate multiple identifiers (e.g., probe sets) mapping to a single gene or molecule in the Global Molecular Network.

In addition, upstream regulator analysis predicted significant inhibition of the estrogen receptor (ER), with a Z-score of −5.8, suggesting altered hormone-related transcriptional regulation in doxorubicin-treated liposarcoma samples (Figure 6).

### 3.4. Canonical Pathways Related to the Liposarcomal Tumor Growth

**Figure 7:**
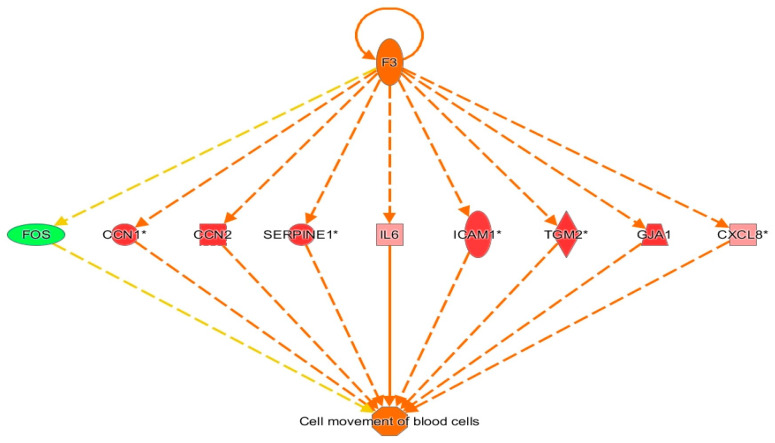
Predicted regulatory effect of gene F3 in liposarcoma, indirectly influencing the movement of blood cells. Different shapes represent molecular classes. Red/green denotes over-/under-expression, and orange denotesactivation. Solid lines show direct interactions; dashed lines represent indirect interactions (*). Asterisks indicate multiple identifiers (e.g., probe sets) mapping to a single gene or molecule in the Global Molecular Network.

Further pathway and network analyses emphasized transcriptional patterns related to liposarcomal tumorigenesis, including immune-related and stromal-associated gene expression patterns. Within these networks, the F3 gene (tissue factor) emerged as a node associated with transcriptional signatures related to blood cell movement, as visualized in Figure 7.

### 3.5. FGF1-Associated Transcriptional Programs

*FGF1* was identified as a prominent regulatory node within the IPA-derived networks. Elevated *FGF1* expression was associated with survival-related, proliferation-related, and anti-apoptotic gene expression patterns, positioning it within adaptive transcriptional programs observed in doxorubicin-treated samples.

### 3.6. In Silico Validation of DE Genes (Further Analysis by Gene Set Enrichment Analysis, (GSEA))

In order to externally validate the DEG list, we compared our results with the RT-qPCR validation that had been conducted by the original contributors of GSE12972 (Daigeler et al., 2008) [10], for which 11 of the genes in our list were experimentally validated and did not yield a correlation coefficient below 0.8, with a Pearson correlation of 0.913 between the comparison of microarray and RT-qPCR results. Of these 11 RT-qPCR-confirmed transcripts, our pipeline captures a transcriptional response that is biologically relevant, as evidenced by the overlap with the programs from Daigeler et al. 2008 [10], which involves *CDKN1A*, *FAS* and *TNFRSF10B*, thus providing experimental validation for this subset. Each of the four upstream regulators identified in this study has extensive prior studies demonstrating associations with anthracycline-induced transcriptional responses, EMT-like states, inflammatory and immune-modulatory programs, chromatin-remodeling-driven plasticity, and survival signaling, respectively. Taken together, these convergent paths of evidence support the biological plausibility of the regulatory programs described here, and we recognize that prospective validation in additional cohorts of liposarcomas, such as through RNA-seq and/or single-cell analyses of pre- and post-treatment samples, is warranted. Gene Set Enrichment Analysis (GSEA), the computational method for the validation of our gene set, was used to determine whether an a priori defined set of genes shows statistically significant, concordant differences among gene patterns upon exposure to Doxorubicin. Supplementary Materials S1–S7.

### 3.7. Differentially Expressed Genes Associated with Immune Processes

Among the DEGs, several immune- and inflammation-related genes were prominently altered. The most significantly upregulated genes, including *AIF1*, *MNDA*, and *CD163*, are listed in Table 2.

Conversely, underexpressed genes such as *CCL20, CXCL8*, and *IL24* are shown in Table 3, reflecting altered chemokine and immune activation-related transcriptional patterns.

## 4. Discussion

This study provides an integrative transcriptomic interpretation of doxorubicin-treated liposarcoma, emphasizing coordinated regulatory adaptation rather than isolated pathway activation. By combining differential gene expression analysis with upstream regulator and network-based inference, the findings suggest a model of multiple parallel regulatory axes during liposarcoma treatment with Doxorubicin, which underlie the versatile transcriptional pathways that arise during chemotherapeutic exposure.

**Figure 8:**
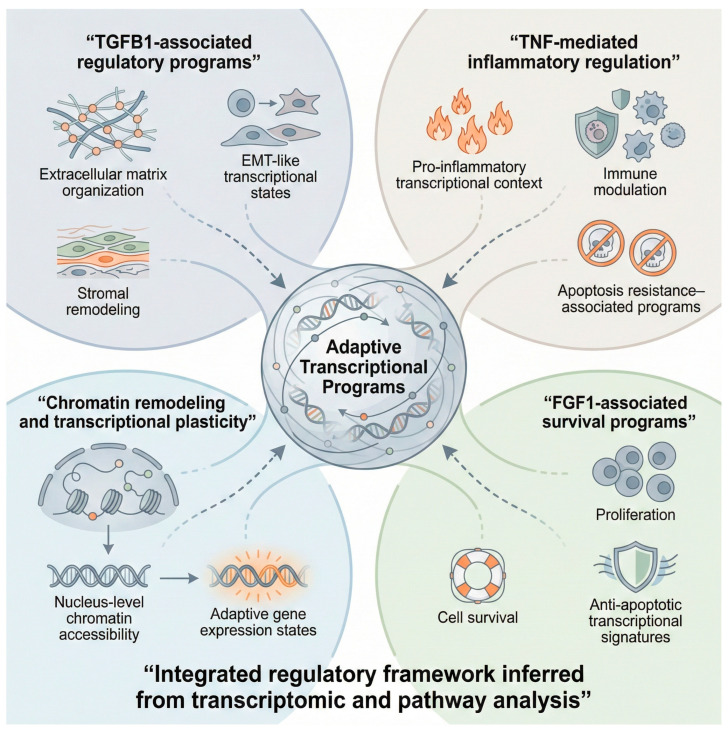
Integrated regulatory framework of adaptive transcriptional changes in doxorubicin-treated liposarcoma. Schematic representation of convergent regulatory patterns inferred from transcriptomic and pathway analyses. *TGFB1*-associated patterns relate to extracellular matrix organization and EMT-like transcriptional states, *TNF*-mediated regulation to inflammatory and immune-modulatory contexts, *SMARCA4*-associated chromatin remodeling to transcriptional plasticity, and *FGF1*-associated programs to survival and anti-apoptotic gene expression. These axes converge on adaptive transcriptional patterns underlying liposarcoma response to chemotherapeutic stress.

A central insight of this work is that doxorubicin-associated transcriptional changes are not adequately explained by a single dominant mechanism. Instead, the data point to the convergence of stromal-associated, inflammatory, chromatin-mediated, and survival-related regulatory pathways and genes during liposarcoma treatment with Doxorubicin, resulting in a unified adaptive transcriptional landscape. This systems-level interpretation is captured in the integrated regulatory framework summarizing the major transcriptional patterns inferred from the analysis (Figure 8).

Within this framework, *TGFB1*-associated transcriptional programs are primarily linked to extracellular matrix organization, stromal remodeling, and EMT-like transcriptional states [11,12]. These processes are known to influence tissue architecture and cellular plasticity, suggesting that *TGFB1*-associated transcriptional activity may be associated with therapeutic responsiveness indirectly by modulating transcriptional context rather than acting as a direct mediator of resistance [13,14].

In parallel, *TNF*-associated inflammatory transcriptional signatures represent a second major component of the adaptive response. The associated gene expression patterns are consistent with immune modulation and apoptosis resistance-associated changes rather than acute inflammatory and specific cytotoxicity [14,15]. This supports the interpretation that inflammatory signaling under chemotherapeutic stress contributes to stabilizing transcriptional states that favor tumor persistence.

Chromatin remodeling, reflected by *SMARCA4*-associated regulatory activity, emerges as a unifying axis linking these diverse transcriptional changes [16]. By facilitating chromatin accessibility and transcriptional plasticity, chromatin-level regulation may enable tumor cells to integrate stromal and inflammatory cues without requiring fixed genetic alterations [17,18]. This regulatory flexibility provides a coherent explanation for the coexistence of heterogeneous transcriptional programs observed in treated tumors.

*FGF1*-associated transcriptional signatures further support this adaptive landscape. This observation aligns with broader signaling contexts in which fibroblast growth factor activity has been linked to downstream engagement of survival pathways, including *PI3K/AKT* signaling, which has been the focus of recent efforts in anticancer inhibitor design and clinical investigation [19]. Rather than functioning as a primary driver, *FGF1*-related transcriptional signatures appear to support cell survival, proliferation, and anti-apoptotic states within the broader regulatory context established by stromal, inflammatory, and chromatin-associated programs, highlighting the cooperative nature of growth factor signaling during chemotherapy exposure [20,21].

Importantly, the regulatory framework proposed here does not represent a mechanistic model of resistance but rather a hypothesis-generating synthesis inferred from transcriptomic and pathway analyses. While the present study focuses on transcriptomic and pathway-level inference, future studies integrating complementary approaches such as metabolic profiling may help further contextualize adaptive transcriptional programs observed under chemotherapeutic stress [22]. By emphasizing coordinated regulatory programs and adaptive transcriptional states, this interpretation offers an alternative lens for understanding therapy-associated adaptation that remains compatible with high-throughput expression data while avoiding overextension into causal molecular claims.

Our multi-layered validation strategy is based on anchoring our bioinformatics-based discoveries to known experimental observations and previous studies on liposarcomas. An important aspect of this validation is the correspondence of our results with the original results described by Daigeler et al. (2008) using the same GSE12972 data set to describe the heterogeneous effects of doxorubicin on primary human liposarcoma cultures [10]. These fundamental liposarcoma-specific observations explicitly cross-reference and confirm our identification of 365 differentially expressed genes (DEGs), and specific engagement of apoptosis and cell-cycle programs including key transcripts *CDKN1A*, *FAS*, and *TNFRSF10B* (TRAIL-R2).

In addition to the internal consistency and genetic/pathway validation, our identified regulatory nodes are also supported by other sarcomatogenic literature. Such *TNF/NFKB* axis activation is placed in the context of earlier reports on anthracycline–NFKB feedback mechanisms in soft-tissue sarcomas, in which it is known to mediate therapeutic response by inducing apoptotic pathways [23]. In the same way, *SMARCA4* was identified as an activated upstream regulator, which is of interest because chromatin-remodeling changes have been observed in undifferentiated and dedifferentiated mesenchymal tumors, reinforcing the idea that *SMARCA4’s* chromatin-remodeling activity contributes to the transcriptional plasticity observed under chemotherapeutic stress. In addition, the correlation of *FGF1* with survival- and anti-apoptotic gene expression patterns is consistent with the known role of the *FGFR* axis in adipocytic differentiation and its functional significance in survival signaling in myxoid liposarcoma [10]. Overall, this validation approach illustrates that although our results are individual bioinformatic products, they are in fact comprehensive, systems-level findings that support known molecular landscapes of Doxorubicin-treated liposarcoma.

All the regulatory programs listed are according to the known molecular biology of liposarcomas. The differential expression of transcripts from the *FGF/FGFR* family that we found is in line with the well-established importance of *FGFR* signaling in the various liposarcoma subtypes, as demonstrated by Wang and colleagues, who showed that FGFR adaptor *FRS2* is amplified and the *FGFR/FRS2* axis is activated in 90–100% of well-differentiated and dedifferentiated liposarcomas, with *FGFR* inhibition challenging liposarcoma cell line growth [24], and by Künstlinger and colleagues, who independently showed that *FGFR2* is overexpressed in myxoid liposarcomas, with *FGFR*-selective inhibitors reducing proliferation, migration, and synergy with trabectedin in patient-derived MLS cell lines [25].

In addition to pathway-level inference, transcriptomic signatures are now beginning to be used as features for machine-learning models to predict drug sensitivity in sarcoma and other cancers with an accuracy of ~70–80% [26,27]. Thus, the upstream-regulator modules reported here may be biologically meaningful modules to use in the future in liposarcoma for the expression-based response classifiers.

From a translational perspective, these findings suggest that targeting isolated molecular pathways may be insufficient to overcome resistance in liposarcoma. Rather than reinforcing reliance on cytotoxic pressure alone, therapeutic strategies that limit regulatory plasticity, constrain chromatin accessibility, or disrupt supportive transcriptional contexts may offer opportunities to improve treatment durability, optimize therapeutic sequencing, or inform alternative treatment approaches beyond conventional chemotherapy. While these implications remain speculative, they underscore the value of systems-level frameworks for guiding future experimental and clinical investigation.

By advancing a systems-level interpretation of doxorubicin-associated adaptation in liposarcoma, this study reframes chemoresistance as the stabilization of adaptive transcriptional programs driven by coordinated regulatory activity. Shifting the analytical focus from individual pathways to integrated transcriptional states provides a conceptual foundation for future experimental validation and for the development of therapeutic strategies aimed at constraining regulatory adaptability. The limitation is the use of a single GSE12972 dataset of 38 arrays, including 19 primary liposarcoma cultures, each profiled as a pair of doxorubicin-treated and vehicle-treated arrays, which has limited statistical power for subtype-stratified inference and precludes formal subgroup analyses. The cohort is also histologically diverse, comprising 7 well-differentiated/atypical lipoma, 4 dedifferentiated lipoma, 4 pleomorphic lipoma, 3 myxoid/round-cell lipoma and 1 myxoid liposarcoma, ranging from grade G1 (*n* = 4) and G2 (*n* = 8) to G3 (*n* = 7) and from primary (*n* = 12) and recurrent (*n* = 6) to metastatic (*n* = 1) tumor origin; although this diversity ensures generalizability across the principal liposarcoma sub-types, it introduces baseline transcriptional heterogeneity that was controlled at the within-culture level but not at the between-subtype level. The treatment condition is a single in vitro exposure (0.5 µg/mL doxorubicin, 24 h, monolayer culture) that reflects only the early acute transcriptional response and not later adaptive states, in vivo pharmacokinetic effects, interactions between combinations of therapies, or an intact tissue microenvironmental context. Another limitation regarding bulk expression measurements from GSE12972 is based on short-term dissociated primary liposarcoma cell cultures, which are grown as a monolayer, and bulk expression cannot distinguish cellular composition. Deconvolution was not performed using deconvolution software including CIBERSORTx and xCell, because their reference signatures (from peripheral blood and intact tissue) have not been validated in short-term sarcoma cultures. Immune- and stromal-related signatures reported here should therefore be interpreted as signatures of gene expression programs present within cultured tumor and co-cultivated stromal cells rather than as an indication of changes in immune infiltration. To determine if these patterns also work at the tissue level in vivo, validation using single-cell or spatial transcriptomics in intact tumor specimens will be needed. Important future steps that they are able to undertake in follow-up studies include broader cross-dataset validation, TCGA-SARC integration and multi-omics extension (proteomic, methylomic, single-cell); however, these are beyond scope of the present transcriptomic synthesis. Regarding current methodological issues and limitations of IPA-based inference, the findings presented here derived from the IPA have some caveats. IPA’s Ingenuity Knowledge Base is compiled from published literature and therefore will have more extensive coverage of well-studied regulators and pathways, and a priori, inferences of upstream regulators may be biased towards extensively characterized ones like *TGFB1* and *TNF* and *SMARCA4*, while the regulators that relate specifically to liposarcoma biology may be underrepresented because literature is sparse. Also, the activation Z-score is a directional consistency score, that is, it measures the consistency of the expression changes observed and curated regulator–target relationships, but it is not a measure of regulator activity. A |Z| ≥ 2 threshold is used to control for the most obvious false positives, but it does not control for confounding from regulators that share the same set of target genes, a relevant concern for the *NF-κB/TNF/TGF-β* axis as many target genes are shared. Finally, IPA assigns a target to one upstream regulator, but in reality, many transcriptional responses are due to a combination of several regulators. Finally, overlap *p*-values provided by IPA are derived from the right-tailed Fisher’s exact test against the Ingenuity Knowledge Base universe and do not reflect the correlation among related regulators and the multiple-testing burden across the entire upstream regulator catalogue. For these reasons, the upstream regulators and pathway calls in this study are emphasized to be hypothesized throughout the manuscript rather than demonstrated. The analyses detailed in results, using an independent transcriptomic data set (GSE2238), a RT-qPCR validation of the original GSE12972 publication, and a set of genes known to be regulated by doxorubicin from the literature, offer further support for the principal regulatory patterns identified (*TNF*-associated inflammatory signaling and stromal/ECM-related transcriptional changes) and partially alleviate concerns of curation-bias. However, nonetheless, formal experimental validation in independent liposarcoma cohorts is needed (orthogonal assays) based on the predicted regulators (ChIP-seq) and paired samples (qPCR or RNA-seq), or on proteomic profiling, before any mechanistic claim can be made. Importantly, the GSE12972 cohort includes all liposarcoma subtypes, which have well-documented differences in chemosensitivity (e.g., myxoid liposarcoma (MLPS), and is classically sensitive to anthracyclines, whereas well-differentiated (WDLPS) and dedifferentiated (DDLPS) liposarcomas are generally chemoresistant); however, the number of samples in each subtype is too imbalanced (*n* = 1 for pure MLPS; *n* = 4 for DDLPS, PLPS and myxoid/round-cell; and *n* = 7 for WDLPS) to allow formal subtype-stratified differential-expression analyses.

Of note, 7 of the 19 primary liposarcoma cultures profiled in GSE12972 dataset were from well-differentiated liposarcoma/atypical lipomatous tumors, pathologically classified as grade 1 (G1) tumors. In the current clinical guidelines, G1 well-differentiated liposarcoma/atypical lipomatous tumors are not treated with systemic chemotherapy (Doxorubicin) because they are inherently chemoresistant and clinically indolent, and these tumors are treated primarily with surgical resection. In the present secondary analysis of this pre-existing data set, the samples were exposed to doxorubicin during in vitro culture, and were thus kept in the present study; however, any doxorubicin-induced transcriptional response observed in this particular G1 subgroup has limited to no direct clinical applicability since the tumors were not treated with the drug during standard clinical care. This point further emphasizes the consideration that such cross-subtype response, described here, should be viewed in the context of liposarcoma biology in general, and not as a clinically actionable predictor of doxorubicin sensitivity by liposarcoma subtype. It is important to note that these transcriptional patterns are a cross-subtype doxorubicin response, not signatures. Adequate size and replication in subtype-pure cohorts will be crucial prior to assigning any of the regulatory patterns described above to a particular liposarcoma subtype and to applying them to subtype-specific therapeutic decisions. Moreover, within-culture paired design assumes that differences in expression are due to the acute (24 h) doxorubicin treatment and not to variability in patient baselines, as each acute doxorubicin-treated profile is compared to its matched vehicle-treated profile from the same primary culture. This resolves the issue of intrinsic heterogeneity: the paired-sample contrast accounts for subject-level baseline differences. The design is not, however, able to model the acquired resistance, which may be due to chronic exposure, clonal selection, or in vivo microenvironmental remodeling, which would require longitudinal clinical paired-sample studies. The signature reported here is therefore best thought of as representing the immediate transcriptional response to doxorubicin in liposarcoma cells and includes potential regulators to be explored in future studies on the development of stable resistance phenotypes from these acute patterns.

## 5. Conclusions

This study provides a comprehensive bioinformatics-driven analysis of transcriptomic alterations associated with doxorubicin treatment in liposarcoma, highlighting coordinated regulatory programs rather than isolated molecular events. Through differential gene expression and pathway-based inference, *TGFB1*, *TNF*, and *SMARCA4* were identified as key upstream regulators shaping transcriptional responses related to extracellular matrix remodeling, inflammatory and immune modulation, and chromatin-mediated transcriptional plasticity. In addition, *FGF1* emerged as a supportive regulatory node associated with survival- and anti-apoptotic gene expression patterns. Collectively, these findings support a systems-level interpretation in which chemotherapeutic exposure is associated with the stabilization of adaptive transcriptional programs involving tumor cells and their liposarcomal tumorigenesis. Rather than implicating a single possible mechanism of resistance, the results emphasize regulatory convergence among stromal, inflammatory, and chromatin-associated processes, offering a coherent framework for understanding transcriptional adaptation in treated liposarcoma. Importantly, this work is intentionally positioned as hypothesis-generating, leveraging transcriptomic and pathway analyses to integrate complex regulatory signals into an interpretable biological model. By shifting the analytical focus from individual pathways to coordinated transcriptional states, the study provides a conceptual foundation for future functional validation, longitudinal profiling, and multi-omics integration. In conclusion, this integrative regulatory framework advances current understanding of doxorubicin-associated adaptation in liposarcoma and may inform future efforts aimed at limiting regulatory plasticity, refining therapeutic strategies, and improving treatment durability in this clinically challenging malignancy.

The regulatory pathways discussed in this work were identified through transcriptomic and pathway-level analyses and have yet to be substantiated in other liposarcoma patient cohorts, both experimentally and clinically, before any mechanistic or therapeutic claims can be made.

## Figures and Tables

**Table 1:** Top 10 upstream regulatory factors identified by Ingenuity Pathway Analysis (IPA).

Upstream Factors Regulator	Type	*p* Value	Z Value
*TGFB1*	growth factor	2.4 × 10^−5^	5.3
*TNF*	cytokine	3.2 × 10^−38^	5.0
*SMARCA4*	transcription regulator	1.3 × 10^−7^	4.6
*EDN1*	cytokine	2.6 × 10^−3^	4.3
*F2*	peptidase	5.6 × 10^−3^	4.3
*TWIST1*	transcription regulator	9.9 × 10^−9^	4.1
*F2R*	G-protein coupled receptor	4.0 × 10^−9^	4.0
lipopolysaccharide	chemical drug	3.0 × 10^−2^	3.9
*NRG1*	growth factor	2.9 × 10^−4^	3.8
*CCR2*	G-protein coupled receptor	5.5 × 10^−4^	3.8

**Table 2:** Top overexpressed genes identified in doxorubicin-treated liposarcoma samples, emphasizing their roles in inflammatory and immune-related processes.

Molecules	Expressed Value	Logfold-Change	*p* Value
*AIF1*	2.879	+2.879	1.01 × 10^−2^
*MNDA*	2.798	+2.798	1.38 × 10^−2^
*CD163*	2.376	+2.376	8.01 × 10^−3^
*SYK*	2.224	+2.224	1.97 × 10^−2^
*P2RY13*	2.112	+2.112	2.10 × 10^−2^
*NCF4*	2.065	+2.065	1.23 × 10^−2^
*TLR7*	2.040	+2.040	3.69 × 10^−2^
*NR5A2*	2.028	+2.028	1.26 × 10^−3^
*MS4A6A*	2.024	+2.024	1.85 × 10^−2^
*KCNQ1*	2.020	+2.020	7.03 × 10^−3^

**Table 3:** Top underexpressed genes identified in doxorubicin-treated liposarcoma samples, with roles in immune activation and chemokine signaling.

Molecules	Expressed Value	Logfold-Change	*p* Value
*CCL20*	−4.545	−4.545	9.10 × 10^−4^
*CXCL8*	−4.046	−4.046	1.36 × 10^−3^
*CXCL5*	−3.801	−3.801	1.27 × 10^−3^
*CXCL3*	−3.562	−3.562	8.15 × 10^−4^
*IL24*	−3.453	−3.453	2.43 × 10^−3^
*PI3*	−3.119	−3.119	7.57 × 10^−4^
*CXCL2*	−3.048	−3.048	5.95 × 10^−3^
*IL6*	−3.032	−3.032	2.67 × 10^−3^
*MMP10*	−3.015	−3.015	9.96 × 10^−4^
*MMP1*	−2.963	−2.963	4.12 × 10^−3^

## Data Availability

Data are contained within the article and Supplementary Materials.

## Supplementary Materials

The following supporting information can be downloaded at: https://www.mdpi.com/article/10.3390/diseases14060219/s1, Gene Set Enrichment Analysis (GSEA), Supplementary Materials S1–S5, External data validation files are summarized in S6 and S7 sheets. S1: MSigDB Compute Overlaps—overexpressed gene panel (gene/geneset overlap matrix). S2: MSigDB Compute Overlaps—overexpressed gene panel (top enriched gene sets table). S3: MSigDB Compute Overlaps—underexpressed gene panel (gene/geneset overlap matrix). S4: MSigDB Compute Overlaps—underexpressed gene panel (top enriched gene sets table). S5: MSigDB_pillar_mapping. S6: validation tables and summary. S7: Independent Data Validation Sheet, raw data file.

## Abbreviations

The following abbreviations are used in this manuscript:

Abbreviation	Full Term
*AIF1*	Allograft Inflammatory Factor 1
*APC*	Article Processing Charge
*BRD4*	Bromodomain Containing 4
*CC BY*	Creative Commons Attribution License
*CCL20*	C-C Motif Chemokine Ligand 20
*CCR2*	C-C Motif Chemokine Receptor 2
*CD163*	Cluster of Differentiation 163
*CXCL2*	C-X-C Motif Chemokine Ligand 2
*CXCL3*	C-X-C Motif Chemokine Ligand 3
*CXCL5*	C-X-C Motif Chemokine Ligand 5
*CXCL8*	C-X-C Motif Chemokine Ligand 8
*DEG*	Differentially Expressed Gene
*DEGs*	Differentially Expressed Genes
*EDN1*	Endothelin 1
*EMT*	Epithelial-to-Mesenchymal Transition
*ER*	Estrogen Receptor
*FDR*	False Discovery Rate
*FGF1*	Fibroblast Growth Factor 1
*FGF*	Fibroblast Growth Factor
*FGFR*	Fibroblast Growth Factor Receptor
*F2*	Coagulation Factor II (Thrombin)
*F2R*	Coagulation Factor II Receptor
*F3*	Coagulation Factor III (Tissue Factor)
GEO	Gene Expression Omnibus
GEO2R	GEO Data Analysis Tool
GSE	Gene Expression Omnibus Series
IPA	Ingenuity Pathway Analysis
*IL6*	Interleukin 6
*IL24*	Interleukin 24
*limma*	Linear Models for Microarray Data
*MMP1*	Matrix Metalloproteinase 1
*MMP10*	Matrix Metalloproteinase 10
*MNDA*	Myeloid Nuclear Differentiation Antigen
*MS4A6A*	Membrane Spanning 4-Domains A6A
*NCBI*	National Center for Biotechnology Information
*NCF4*	Neutrophil Cytosolic Factor 4
*NRG1*	Neuregulin 1
*NR5A2*	Nuclear Receptor Subfamily 5 Group A Member 2
*PI3*	Peptidase Inhibitor 3
*P2RY13*	Purinergic Receptor P2Y13
*SMARCA4*	SWI/SNF Related, Matrix Associated, Actin Dependent Regulator of Chromatin Subfamily A Member 4
*SYK*	Spleen Associated Tyrosine Kinase
*TGFB1*	Transforming Growth Factor Beta 1
*TGF-β*	Transforming Growth Factor Beta
*TLR7*	Toll-Like Receptor 7
*TNF*	Tumor Necrosis Factor
*TWIST1*	Twist Family BHLH Transcription Factor 1
