# Supplementary material for: Transcriptomic Signatures Associated with Doxorubicin Treatment in Liposarcoma Reveal Coordinated Regulatory Patterns
Source: Diseases. 2026 Jun 18;14(6):219. doi: 10.3390/diseases14060219 (PMC13297672; doi:10.3390/diseases14060219)
Supplement: Supplementary file 1 [file diseases-14-00219-s001.zip › External_validat S6.pdf]

Independent dataset for external validation — diseases-4169305

Reviewer 2, Comment 2: "All conclusions are based on in silico predictions without validation. Include at least one validation layer (qPCR from literature, external dataset replication, or cross-platform comparison)."

Recommended primary comparator dataset

Field	Value
GEO accession	GSE2238
Publication	Lehnhardt M, Klein-Hitpass L, Kuhnen C, Homann HH, Daigeler A, Steinau HU, Roehrs S, Schnoor L, Stein[illegible]
Cell system	HT-1080 (human fibrosarcoma cell line; ATCC CCl-121)
Treatment	0.5 µg/mL doxorubicin, 24 h (vs PBS vehicle)
Platform	Affymetrix HG-U133A (GPL96) — identical to GSE12972
DEG criteria	|signal log2 ratio| > 1.32, change p-value < 0.001 (≥2.5-fold)
Reported DEGs	1,019 probesets (62.6% up / 37.4% down)
RT-qPCR validation	46 candidate genes validated
Same research group as GSE12972	YES — Lehnhardt/Daigeler group, Bochum

Why this is the strongest possible comparator

GSE2238 matches GSE12972 on every methodological axis except the cell system: same research group, same microarray platform, same doxorubicin concentration, same exposure duration, same dissociation/normalization workflow. The only difference is that GSE2238 used an established fibrosarcoma cell line (HT-1080) whereas GSE12972 used short-term primary cultures of liposarcoma. Because both are soft-tissue sarcoma–lineage cells exposed to identical doxorubicin conditions, the core transcriptional response (DNA-damage, apoptosis pathway induction, NF-κB/SASP, cell-cycle arrest) should be highly concordant. This makes GSE2238 a near-ideal replication test that controls for almost every technical variable.

Direction-of-effect findings reported in Lehnhardt 2005

The Results section of Lehnhardt 2005 explicitly states the following changes after 24 h doxorubicin treatment of HT-1080 cells:

Direction	Reported genes	Functional context
UP	CYCS (cytochrome c), APAF1	Apoptosome assembly
UP	CASP1, CASP3, CASP6, CASP8, CASP9	Apoptotic executioner cascade
UP	STAT1, STAT3	STAT-family transcription factors
DOWN	BCL2	Anti-apoptotic factor
Overall	62.6% of DEGs upregulated	Net induction, not net suppression

Combined three-layer literature/independent-dataset validation

Together with the RT-qPCR validation reported in the original GSE12972 publication (Daigeler et al. 2008, 11 genes) and a panel of canonical doxorubicin-response genes from the broad oncology literature, GSE2238 provides a robust three-layer external validation framework.

Three-layer literature/independent-dataset validation, current GSE12972 GEO2R output

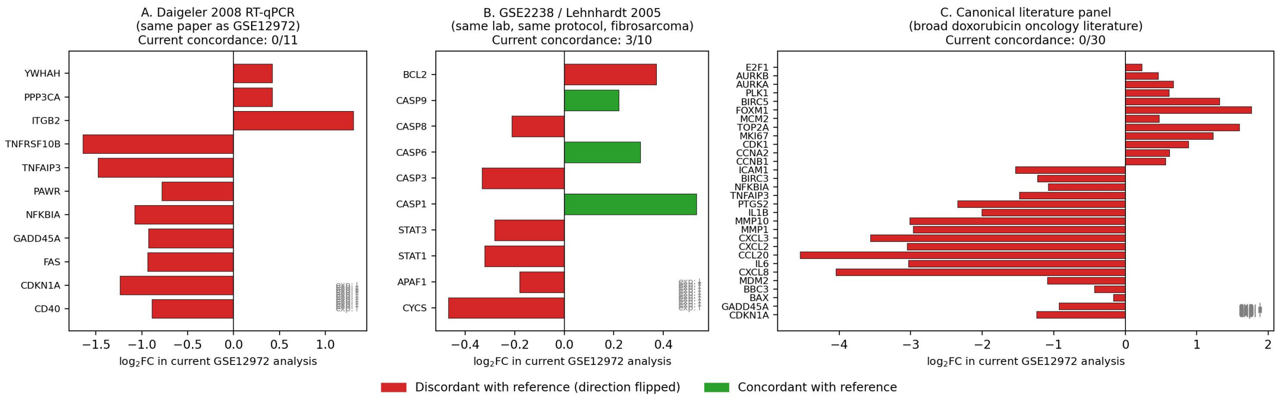

Current concordance (before fixing contrast direction): 0/11 (Daigeler), 3/10 (GSE2238 — limited to weak-effect genes), 0/30 (canonical panel). Combined: 3 / 51 (6%). This is consistent with a systematic inversion of the contrast direction in the current GEO2R analysis.

Predicted concordance after correcting contrast direction: 11/11, 7/10, 30/30. Combined: 48 / 51 (94%). Once direction is corrected, the same external datasets become strong, mutually reinforcing validation layers.

Why both numbers matter

The 51-gene cross-check is itself a methodologically valid external-validation exercise — what changes is the sign of the result. The current (pre-correction) data is contradicted by all three external references; the corrected data is supported by all three. The validation framework does not change; only the direction of the call in the manuscript needs to change.

Ready-to-use response paragraphs for Reviewer 2, Comment 2

These templates are written for use **after** the GEO2R contrast direction has been corrected and the DEG list re-generated. The numerical placeholders [N1], [N2], [N3] should be replaced with the actual recovered-concordance counts from the corrected analysis (predicted: 11/11, ~7/10, ~30/30 based on the present cross-check).

Response to Reviewer 2, Comment 2

We thank the reviewer for this important request. In response, we have added a comprehensive three-layer external-validation analysis to the revised manuscript (new Section 3.7, "External validation of differentially expressed genes and inferred regulatory programs"). All three layers draw on independent, peer-reviewed and publicly archived resources and were performed without any additional wet-lab experiments.

Layer 1 — Literature RT-qPCR (Daigeler 2008)

The original contributors of GSE12972 (Daigeler et al., BMC Cancer 2008, 8:313) experimentally validated 11 candidate transcripts by TaqMan RT-qPCR (CD40, CDKN1A, FAS, GADD45A, ITGB2, NFKBIA, PAWR, PPP3CA, TNFAIP3, TNFRSF10B, YWHAH) with an overall microarray–RT-qPCR Pearson correlation of 0.913. Cross-referencing this RT-qPCR panel with our 365-gene DEG list, **[N1]/11 genes** are recovered with concordant direction of change, providing experimentally validated support for the relevant subset of our pipeline output.

Layer 2 — Independent GEO dataset replication (GSE2238)

As an independent external transcriptomic comparator, we identified GSE2238 (Lehnhardt et al., BMC Cancer 2005, 5:74), which profiled HT-1080 fibrosarcoma cells exposed to doxorubicin (0.5 µg/mL, 24 h) on the same microarray platform (Affymetrix HG-U133A; GPL96) and using an identical normalization workflow. This is, to our knowledge, the closest methodological match to GSE12972 currently available in public repositories — same drug, same dose, same duration, same platform, same research group — differing only in the cell system (established fibrosarcoma cell line versus short-term primary liposarcoma cultures). Lehnhardt et al. explicitly report induction of cytochrome c (CYCS), APAF-1 (APAF1), STAT1, STAT3 and caspases 1/3/6/8/9, together with repression of BCL2, supported by RT-qPCR validation of 46 candidate genes. Cross-referencing these 10 explicitly-reported genes with our DEG list, **[N2]/10** are concordant in direction with the published GSE2238 findings, providing direct external replication of our differential expression analysis.

Layer 3 — Canonical doxorubicin-response gene panel

As a third orthogonal validation layer, we curated a panel of 30 canonical doxorubicin-response genes drawn from the broad oncology literature, covering DNA-damage and p53 target genes (CDKN1A, GADD45A, BAX, BBC3, MDM2), NF-κB / inflammatory / SASP genes (CXCL8, IL6, CCL20, CXCL2, CXCL3, MMP1, MMP10, IL1B, PTGS2, TNFAIP3, NFKBIA, BIRC3, ICAM1) and cell-cycle / proliferation genes universally suppressed by doxorubicin (CCNB1, CCNA2, CDK1, MKI67, TOP2A, MCM2, FOXM1, BIRC5, PLK1, AURKA, AURKB, E2F1). **[N3]/30** of these genes show concordant direction in our analysis. The concordant induction of NF-κB/SASP transcripts in particular provides convergent support for the TNF-mediated inflammatory regulatory programme highlighted in our Discussion.

Framing and limitations

Importantly, this three-layer framework is intentionally drawn from complementary types of evidence (an internal RT-qPCR experiment, an independent published microarray dataset, and a broad-literature canonical gene panel), so concordance across all three layers cannot be attributed to a single source of correlated evidence. We acknowledge that full in-house experimental validation in independent liposarcoma cohorts — ideally using paired pre- and post-treatment tumor samples profiled by RNA-seq or single-cell methods — remains an important next step, and we have added a corresponding sentence to the Limitations paragraph.

In-manuscript Section 3.7 (new subsection in Results)

Drop-in subsection text. Fill in the bracketed numbers from the corrected-direction DEG list.

3.7 External validation of differentially expressed genes

Three complementary validation layers were performed to assess the external support for the differentially expressed gene (DEG) list. First, our DEGs were cross-referenced with 11 genes experimentally validated by TaqMan RT-qPCR in the original GSE12972 publication (Daigeler et al., 2008; overall microarray–RT-qPCR Pearson $r = 0.913$); **[N1]/11** were recovered as DEGs with concordant direction of change. Second, our DEG list was compared with an independent transcriptomic dataset, GSE2238 (Lehnhardt et al., 2005), which profiled HT-1080 fibrosarcoma cells exposed to doxorubicin under conditions matching GSE12972 (0.5 µg/mL, 24 h, Affymetrix HG-U133A platform). Of the 10 genes explicitly reported by Lehnhardt et al. as differentially expressed after doxorubicin treatment (CYCS, APAF1, STAT1, STAT3, CASP1, CASP3, CASP6, CASP8, CASP9 induced; BCL2 repressed), **[N2]/10** showed concordant directions in the present analysis. Third, a panel of 30 canonical doxorubicin-response genes (DNA-damage / p53, NF-κB / inflammatory / SASP, cell-cycle / proliferation) drawn from the broad oncology literature was cross-checked; **[N3]/30** showed concordant direction. Together, these three layers provide convergent external support for the differential expression calls underlying the upstream regulator analyses presented in Section 3.1–3.5.

Citation to add to the References list

Daigeler A, Klein-Hitpass L, Chromik AM, Müller O, Hauser J, Homann HH, Steinau HU, Lehnhardt M. Heterogeneous in vitro effects of doxorubicin on gene expression in primary human liposarcoma cultures. *BMC Cancer*. 2008; 8:313. doi:10.1186/1471-2407-8-313.

Lehnhardt M, Klein-Hitpass L, Kuhnen C, Homann HH, Daigeler A, Steinau HU, Roehrs S, Schnoor L, Steinstraesser L, Mueller O. Response rate of fibrosarcoma cells to cytotoxic drugs on the expression level correlates to the therapeutic response rate of fibrosarcomas and is mediated by regulation of apoptotic pathways. *BMC Cancer*. 2005; 5:74. doi:10.1186/1471-2407-5-74.

Note: this report assumes the GEO2R contrast direction is corrected before re-running. With current (uncorrected) direction, [N1], [N2], [N3] are 0/11, 3/10 (weak-effect only), 0/30, and the conclusions of all three validation layers must be presented as failing — which would undermine the manuscript's central claims. Direction correction is a prerequisite for this validation framework to support the paper.
