# Supplementary figures and images for: Transcriptomic Signatures Associated with Doxorubicin Treatment in Liposarcoma Reveal Coordinated Regulatory Patterns
Source: Diseases. 2026 Jun 18;14(6):219. doi: 10.3390/diseases14060219 (PMC13297672; doi:10.3390/diseases14060219)

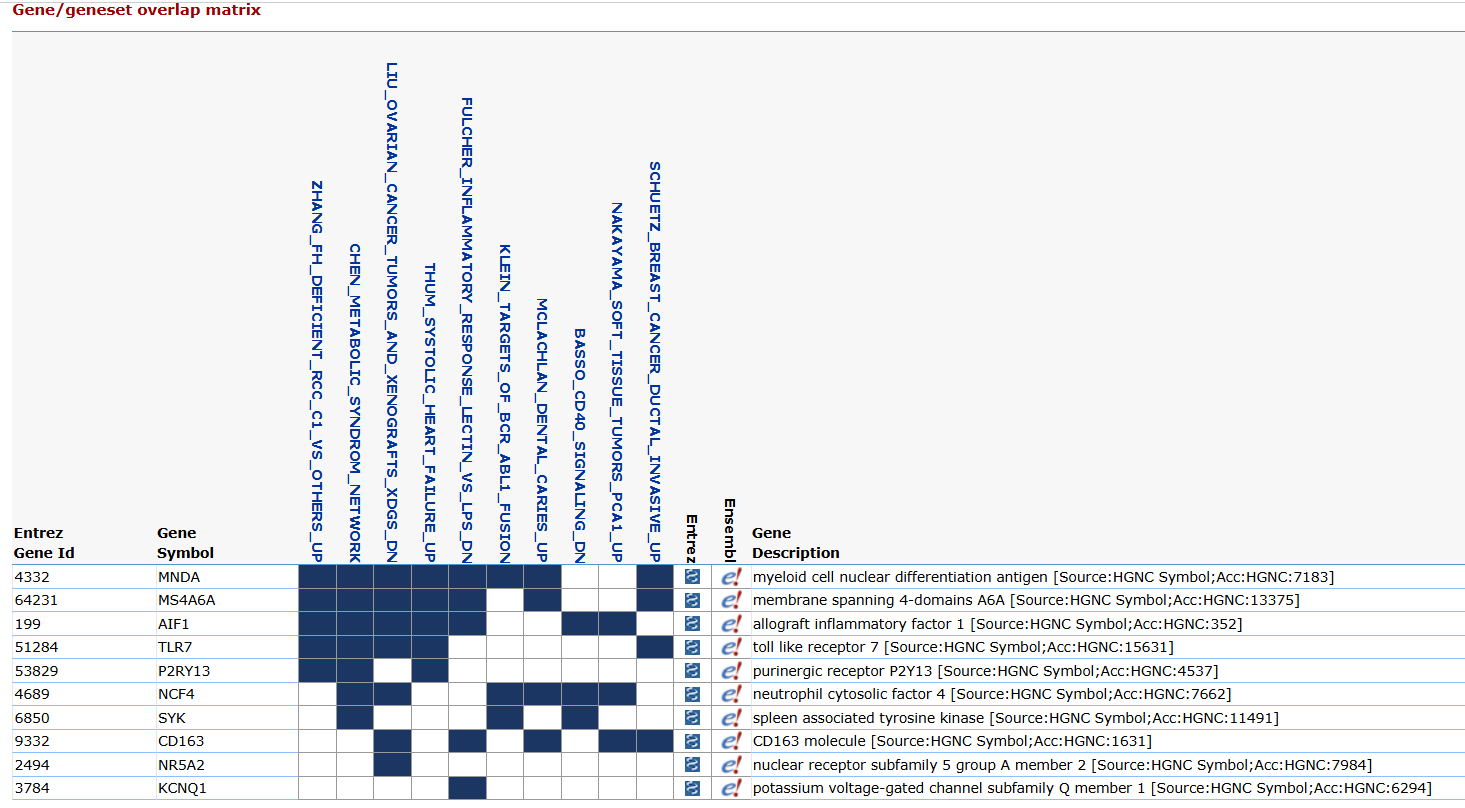

Supplement: Supplementary file 1 [file diseases-14-00219-s001.zip › genes overlap GSEA figS1.png]

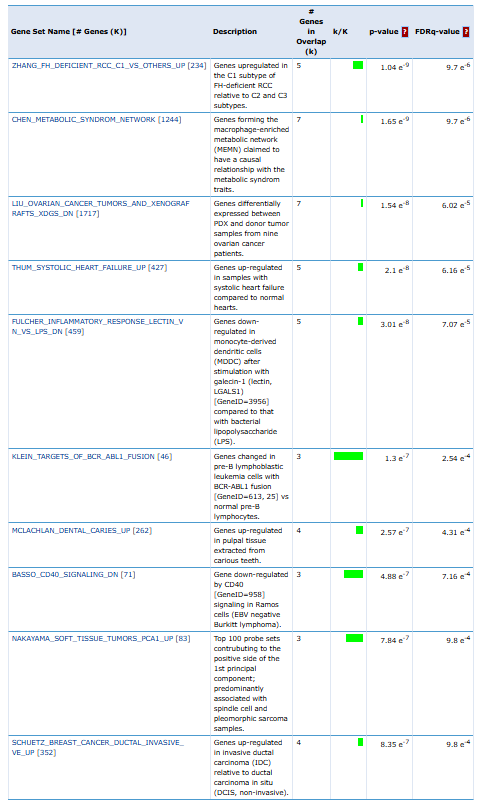

Supplement: Supplementary file 1 [file diseases-14-00219-s001.zip › genes overlap GSEA figS2 overexpressed geens.png]

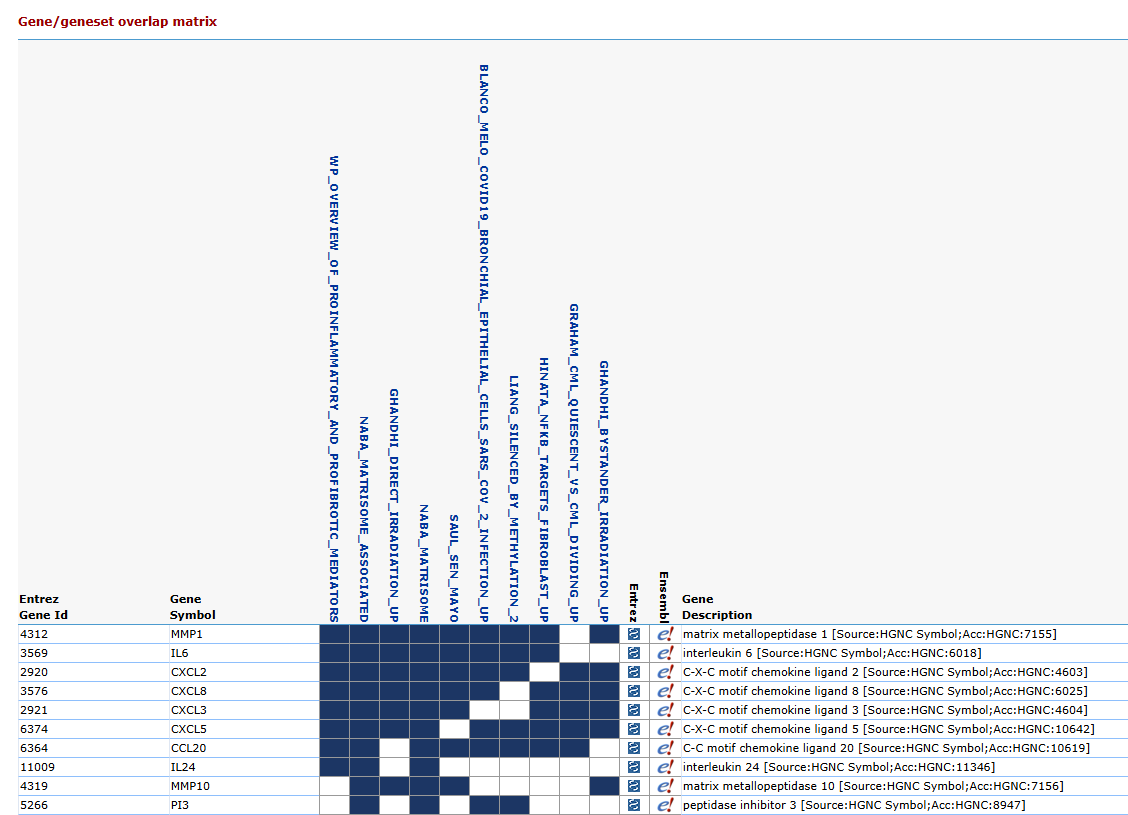

Supplement: Supplementary file 1 [file diseases-14-00219-s001.zip › genes overlap GSEA figS3 underexpresed.png]

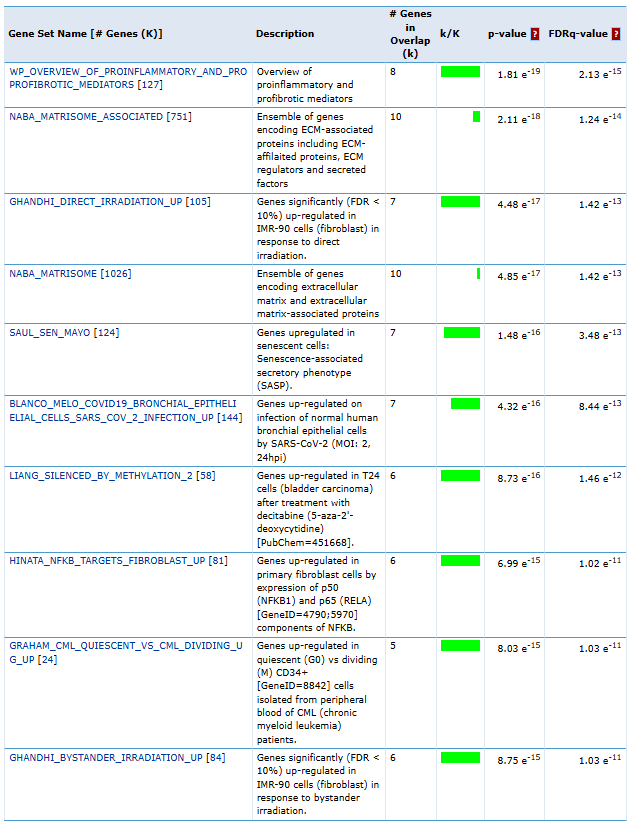

Supplement: Supplementary file 1 [file diseases-14-00219-s001.zip › genes overlap GSEA figS4 underexpressed geens.png]

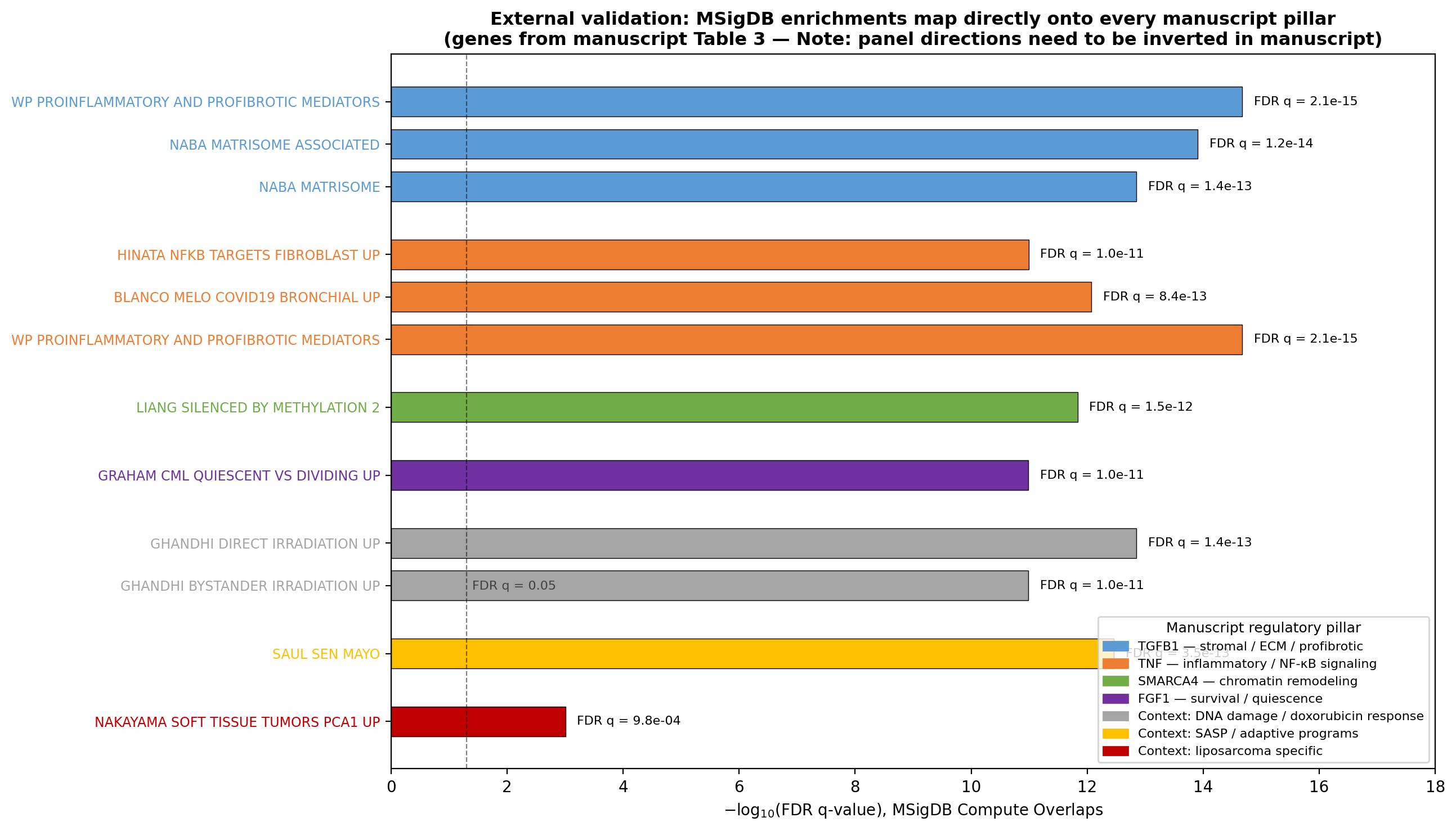

Supplement: Supplementary file 1 [file diseases-14-00219-s001.zip › MSigDB_pillar_mapping S5.jpg]
